# Global transcriptome analysis identifies critical functional modules associated with multiple abiotic stress responses in microalgae *Chromochloris zofingiensis*

**DOI:** 10.1371/journal.pone.0307248

**Published:** 2024-08-22

**Authors:** Bahman Panahi

**Affiliations:** Department of Genomics, Branch for Northwest & West Region, Agricultural Biotechnology Research Institute of Iran (ABRII), Agricultural Research, Education and Extension Organization (AREEO), Tabriz, Iran; Sathyabama Institute of Science and Technology, INDIA

## Abstract

In the current study, systems biology approach was applied to get a deep insight regarding the regulatory mechanisms of *Chromochloris zofingiensis* under overall stress conditions. Meta-analysis was performed using p-values combination of differentially expressed genes. To identify the informative models related to stress conditions, two distinct weighted gene co-expression networks were constructed and preservation analyses were performed using medianRankand Zsummary algorithms. Moreover, functional enrichment analysis of non-preserved modules was performed to shed light on the biological performance of underlying genes in the non-preserved modules. In the next step, the gene regulatory networks between top hub genes of non-preserved modules and transcription factors were inferred using ensemble of trees algorithm. Results showed that the power of beta = 7 was the best soft-thresholding value to ensure a scale-free network, leading to the determination of 12 co-expression modules with an average size of 128 genes. Preservation analysis showed that the connectivity pattern of the six modules including the blue, black, yellow, pink, greenyellow, and turquoise changed during stress condition which defined as non-preserved modules. Examples of enriched pathways in non-preserved modules were Oxidative phosphorylation”, “Vitamin B6 metabolism”, and “Arachidonic acid metabolism”. Constructed regulatory network between identified TFs and top hub genes of non-preserved module such as Cz06g10250, Cz03g12130 showed that some specific TFs such as C3H and SQUAMOSA promoter binding protein (SBP) specifically regulates the specific hubs. The current findings add substantially to our understanding of the stress responsive underlying mechanism of *C*. *zofingiensis* for future studies and metabolite production programs.

## Introduction

Human population growth leads to problems such as climate change and food and energy shortages. Microalgae have the great potential to become a sustainable resource for biofuels and bio pigments, because they have important advantages such as efficient CO2 fixation using solar energy, higher growth rate, and independence of cultivation systems to arable land and water [1, 2]. Despite this, the production of bio pigments such as carotenoids and lipids from microalgae is limited by practical problems, resulting in low productivity and high costs [3].

Microalgae, including *Chromochloris zofingiensis*, can experience various stress conditions, such as nutrient limitation, high or low temperatures, salinity fluctuations, and oxidative stress. These stressors can affect the growth, metabolism, and overall physiological state of the microalgae. Under stress conditions, microalgae activate specific genes involved in stress response pathways. These genes may code for proteins that help the cells survive and adapt to the stressor [4]. Moreover, these organisms synthesize protective molecules such as antioxidants, osmoprotectants, and heat shock proteins to counteract the effects of stress. These molecules help maintain cellular homeostasis and protect cellular structures from damage. Additionally, it undergoes metabolic adjustments to optimize energy utilization and maintain essential cellular functions. This involves changes in the production of metabolites, lipid composition, and energy storage. On the other hand, Since *C*. *zofingiensis* is a photosynthetic microalga; it undergoes changes in photosynthetic activity in response to different stress. This could involve alterations in chlorophyll content, photosystem efficiency, and carbon assimilation [5].

It has been demonstrated that the CO2 fixation efficiency and consequently the lipids and other metabolites accumulation are changed by growth environmental modulations with different stressors and medium formulations [4, 6]. Microalgae respond rapidly to environmental stressors such as nutrient depletion/repletion, salt stress, sulfur, and phosphorous starvation, and intense light conditions, leading to carbon donation for metabolite biosynthesis and accumulation [7]. Therefore, this strategy is among the most promising approaches for improving the productivity rate of microalgae-driven metabolites. Nevertheless, progress in this regard has been limited due to the lack of the underlying responsive mechanisms and subsequent functional impacts on their metabolic pathways [8].

Prior studies have dissected the stress-responsive mechanisms of microalgae in metabolites accumulation conditions, for example, integration of meta-analysis of transcriptome data highlighted the cross-talk between Ca2^+^ signal transduction, lipid accumulation, and ROS signaling network in salt stress conditions [4]; however, underlying molecular mechanisms are not well known to efficiently manipulate for new strain development, mainly due to the most of the studies focus only on the identification and screening of differentially expressed genes and connectivity and systems level analysis were not well considered [9]. It is whilst that the co-expression based network analysis in the context of the transcriptome information provides valuable knowledge regarding the systems level behavior of differentially expressed genes under a specific condition, which cannot be detected by standard transcriptome and network analysis [10–12].

*Chromochloris zofingiensis* also referred to *Muriella zofingiensis* is a freshwater eukaryotic green microalga that belongs to the Chlorophyceae group with robust performance in growth under photoautotrophic, heterotrophic, and mixotrophic conditions. Moreover, *C*. *zofingiensis*, concurrently accumulate value-added compounds such as ketocarotenoid astaxanthin and neutral lipid triacylglycerol (TAG), which makes it an appealing alga for integrated production of the aforementioned compounds at commercial scale [13–15].

A major effort has been dedicated to optimizing the culture conditions to enhance *C*. *zofingiensis* production [16]. However, manipulation of the genetics potential is still needed to improve the production rate of different bio pigments [17]. These will rely on understanding the transcriptional regulation at system levels especially under over-accumulation conditions.

The development of next-generation sequencing technology provides an unprecedented chance for the dissection of complex biological systems [4, 18, 19]. Regarding C. *zofingiensis*, different transcriptomic studies based on RNA sequencing technology have been performed and provide some insight into different stressors related pathways. However, generalization of the major transcriptome regulatory mechanism is essential to provide meaningful and precise biological conclusions [12]. Integrative investigation of transcriptome data using meta-analysis and system-level network analysis is among the most promising strategies put forth to overcome this challenge [3, 20–22]. Therefore, in the current study we harnessed an integrative systems biology approach to the analysis of transcriptome information of C. *zofingiensis* to get a deep insight regarding the regulatory mechanisms under overall stress conditions.

## Methods and material

### Gene expression datasets

The RNA-Seq datasets related to stress treatments including salinity, light stress, sulfur starvation and depletion, nitrogen deprivation, H_2_O_2_ treatment, iron depletion, phosphorous depletion, Rose Bengal treatment, anaerobic condition, and glucose treatment were retrieved from the Gene Expression Omnibus (GEO) (https://www.ncbi.nlm.nih.gov/geo/) and Sequence Read Archive (SRA) (https://www.ncbi.nlm.nih.gov/sra) databases. Four RNA-Seq datasets related to stress-responsive transcriptome were included in our study.

The first dataset (GSE92514) covers RNA-Seq data of *C*. *zofingiensis* following the transition from medium light to high light. Cultures of *Chromochloris zofingiensis* were grown diurnally (16 h light, 8 h dark) in 100 μmol photons m^−2 s^−1 medium light (ML). At t = 0, cultures were transferred to 400 μmol photons m^−2 s^−1 high light (HL). Samples were collected in quadruplicate at 0, 0.5, 1, 3, 6, and 12 h for ML cultures and at 0.5, 1, 3, 6, and 12 h for HL cultures [23]. The second dataset (GSE113802) includes 21 samples of RNA-Seq profiling of C. *zofingiensis* in response to nitrogen deprivation. In this data set, whole-cell mRNA profiling was performed at 5-time points (0, 3, 6, 12, and 24 h) of nitrogen deprivation, in triplicate [15]. The third data set (GSE125419) surveyed the transcriptomic changes induced by salinity stress. In this data set, which had 6 samples, cells were harvested in the exponential phase and re-inoculated in Kuhl medium containing 200 mM of NaCl for salinity stress. Then, samples were collected for RNA isolation at 0 h and 6 h after re-inoculation with three biological replicates under each condition (salinity stress and control) c The fourth data set (GSE130454) were performed to time-resolved transcriptome analysis under sulfur starvation stress. In this experiment, containing 18 samples, cells in the exponential phase were harvested and re-inoculated in a modified Kuhl medium in which MgSO4 was replaced with MgCl2 for sulfur starvation stress. Then, treated samples were collected for RNA isolation at 0 h and 6, 12, 24, and 48 h after re-inoculation. After 48 h of sulfur starvation, cells were re-inoculation into fresh Kuhl medium for sulfur replenishment and samples were collected after 12 h. Three biological replicates of algal cultures were carried out [15].

### RNA-Seq data analysis

Raw fastq files of the aforementioned datasets were retrieved and the quality of the short reads was surveyed using FastQC software version 0.11.5 [24]. Then, low-quality reads (quality score below 30) were excluded from data using Trimmomatic software version 0.32 [25]. The criteria for trimming of raw sequences were as follows: LEADING: 30, TRAILING: 3, SLIDINGWINDOW: 4:20, and MINLEN: 45. The paired-end clean reads that passed quality filters were aligned to reference genome *C*. *zofingiensis* v5.2.3.2 [23] using TopHat v2.0.12 [26] was performed. To count the reads numbers mapped to each gene, HT-seq software were used. The differential expression of genes between stress treated and control samples was estimated by using the DESeq2 package [27]. A significant differential expression was defined as a fold change ≥ |1| and a False Discovery Rate (FDR) corrected *P-value* ≤ 0.05 [28].

### Meta-analysis of expression data

Meta-analysis was performed using p-values combination of differentially expressed genes combined with Fisher normalization algorithm complemented in metaRNASeq R package [29]. In this step, stress treated dataset was analyzed separately using the GLM (Generalized Linear Models). Fisher’s method combines the *P* -values from each experiment with one test statistic defined as:

$$X^{2}=-2\sum_{s=1}^{S}ln(p_{gs})$$

where *p*_*i*_ denotes the raw *P*-value obtained from gene *g* in experiment *s*. *S* is the number of the combined experiments. Under the null hypothesis, the test statistic *χ* follows a χ^2^ distribution with 2 *S* degrees of freedom. This test provides a meta *P*-value, and the classical procedures for multiple testing correction can be applied to obtain the adjusted *P*-values to control the false discovery rate.

### Weighted gene co-expression network analysis

Expression values of meta-genes provided by the direct merging approach, as prescribed in [10, 11, 30], were normalized using variance StabilizingTransformation (vst) function. In the next step, the correlation of meta genes was determined with Pearson’s correlation algorithm. Then, created similarity matrix was transformed into an adjacency matrix using the following formula:

$$a_{ij}={\left(0.5*\left(1+cor\left(i,j\right)\right)\right)}^{ᵝ}$$

Where *a*_*ij*_ represent the adjacencies between genes as a connection strengths index.

The adjacency was transformed into a topological overlap matrix (TOM) and corresponding dissimilarity matrix (1 − *TOM*) using the following equation

$${TOM}_{i,j}=\frac{\sum_{u}a_{iu}\;a_{uj}+a_{ij}}{\text{min}\left(k_{i},k_{j}\right)+1-a_{ij}},\;K_{i}=\sum_{u}a_{iu}$$

Where row index *u*(*u* = 1, …, *m*) represents the sample measurements.

Finally, average linkage hierarchical clustering analysis was performed by the topological overlap-based dissimilarity matrix as input, and modules were determined by a dynamic hybrid tree cutting algorithm implemented in the WGCNA R package.

### Module preservation analysis

To identify the informative models related to stress conditions, two distinct co-expression networks were constructed and preservation analyses were performed using the preservation function implemented in WGCNA package version 1.72–5 [31]. The preservation status of defined co-expressed modules of control networks was tested in the stress condition based on medianRankand Zsummary algorithms as described in [12]. Zsummary combines multiple statistics into a single overall measure of preservation that considers density and connectivity aspects of preservation. The higher Zsummary value indicates the strong preservation of the co-expression module in both control and stress conditions. Median rank is another algorithm which analysis the preservation of the module but in contrary with the Zsummary, it is the size independent algorithm, and lower value of a medianRankshown the strong preservation in control and stress conditions. To survey the statistical significance, preservation analysis was performed using permutation = 200. Zsummary < 10 or median Rank > 8 were used as criteria for the selection of non-preserved modules. Non-preserved modules as informative modules were selected for further functional and regulatory network analysis.

### Inferring gene regulatory networks

Transcription factor (TF) annotations for the *C*. *zofingiensis* genome were compiled based on proteome homology studies compiled as described by [32]. We obtained the protein IDs of predicted *C*. *zofingiensis* TFs from [33]. In the next step, the gene regulatory networks between top hub genes of non-preserved modules and identified TFs were inferred using ensemble of trees algorithm implemented in GENIE3 package version 3.19 [34].

### Functional enrichment analysis

To survey of the functional impacts of significant modules, enrichment analysis was performed based on gene ontology (GO) and the Kyoto encyclopedia of genes and genome (KEGG) pathway as prescribed by [35]. AgriGO v2.0 (http://systemsbiology.cpolar.cn/agriGOv2/) is primarily used for performing GO enrichment analysis. This involves identifying which GO terms (biological processes, molecular functions, and cellular components) are significantly overrepresented in a given set of genes. Pathway enrichment was also carried out using Algal Functional Annotation Tool (http://pathways.mcdb.ucla.edu/algal/index.html) by setting P-value < 0.05 as a cut-off criterion. Algal Functional Annotation Tool includes features for pathway enrichment analysis to understand the involvement of genes in specific metabolic and signaling pathways.

For gene ontology enrichment of the gene list of non-preserved modules mapped to its corresponding GO terms based on reported annotations. In the next step, the occurrence of the annotated GOs were compared with the reference genome annotations. Finally, Fisher’s exact test was performed to determine if a GO term is significantly overrepresented in the input list compared to the background set.

Identifying overrepresented biological pathways in a group of genes provides valuable insights into fundamental biological mechanisms. Enrichment analysis is used to interpret large-scale omics data, including genomics, transcriptomics, and proteomics, enabling researchers to generate hypotheses about the biological significance of observed gene expression patterns. Functional enrichment analysis is an essential tool in biological research, revealing the functional landscape of genes and their interactions. This knowledge is crucial for advancing our understanding of complex biological processes, such as stress-responsive mechanisms.

### Determination and validation of hub genes

In complex biological networks such as a response to a different physiological condition, the genes with the highest degree of connectivity in the systems level networks were considered essential genes with significant importance related to the studied biological process [36, 37]. Hub genes are central to the regulation of complex gene interaction networks. Studying hub genes provide valuable knowledge for understanding how regulatory networks are organized and how they control gene expression and cellular functions. Moreover, Hub genes coordinate multiple biological processes, acting as central nodes that integrate various signaling and regulatory pathways. Based on this fact, hub genes in significant co-expressed modules were determined according to module membership (MM) and eigengene-based module connectivity (kME). Genes with higher intramodular connectivity were considered hub genes in the significant module. Subsequently, determined hub genes were further validated using leave-one-out cross-validation (LOOCV) algorithm implemented.in R *Package Caret* V.6.0–84 [38]. Moreover, protein-protein interaction (PPI) networks of stress related significant modules were constructed using STRING database through homologous proteins interactions of *Chlamydomonas reinhardtii*. For construction of PPI network, gene neighborhood, gene fusion, and text mining algorithms implemented in STRING data base were harnessed and visualized using Cytoscape Version 3.7.2 [39].

## Results and discussion

### RNA-seq data analysis

**Fig 1:**
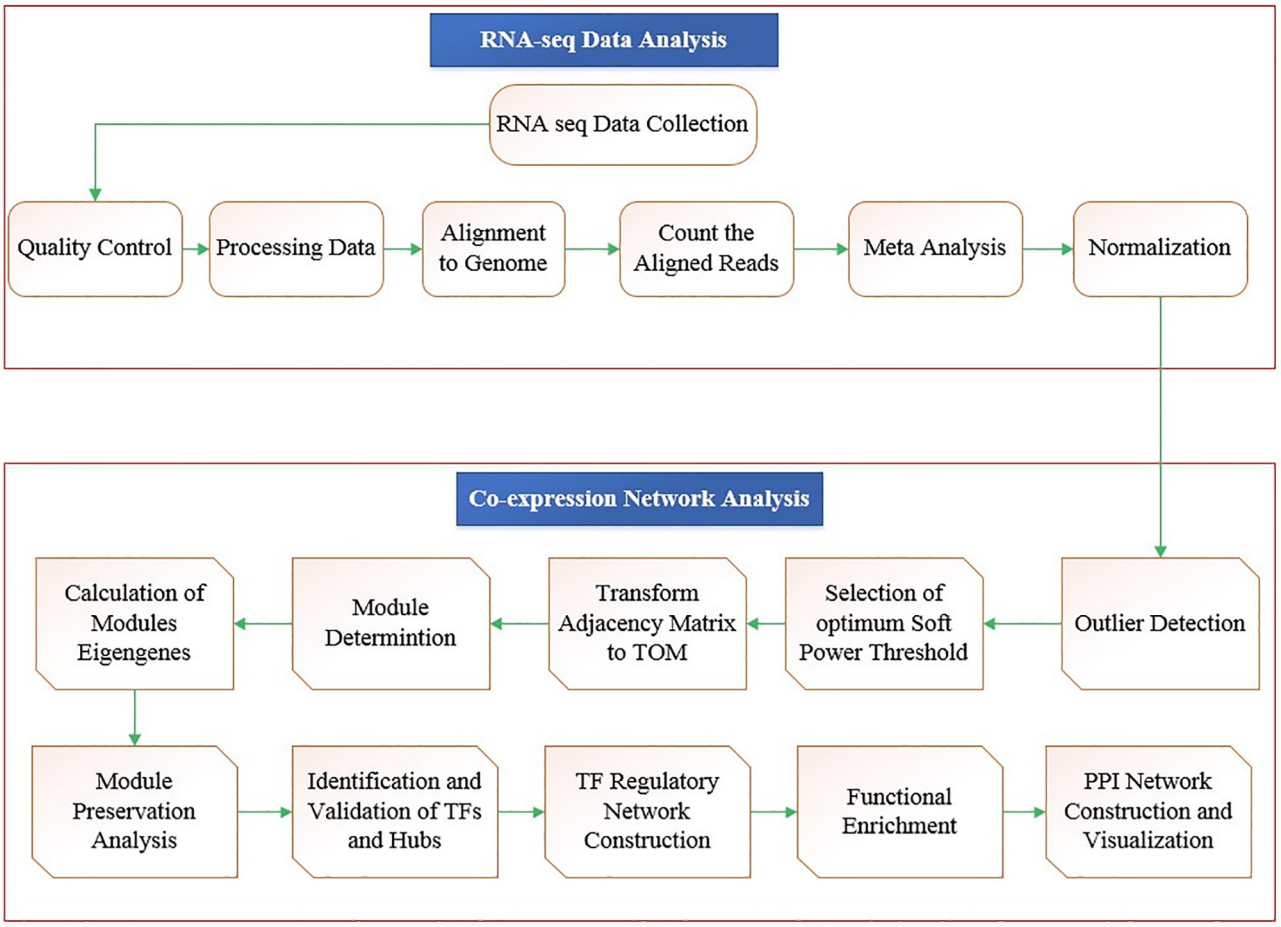
Step-by- step workflow of applied pipeline in current study.

The step-by-step workflow of the RNA-seq data analysis, meta-analysis, and co-expression network analysis used in this study is presented in Fig 1. Briefly, collected RNA-seq data sets contained 75 samples of which 57 were related to stress treated samples and 18 samples were among the control samples. After normalization, filtering of genes with low variance, and meta-analysis, the final matrix had 1654 genes which were used for co-expression network construction and further analysis (S1 Table).

### Weighted gene co-expression network construction

**Fig 2:**
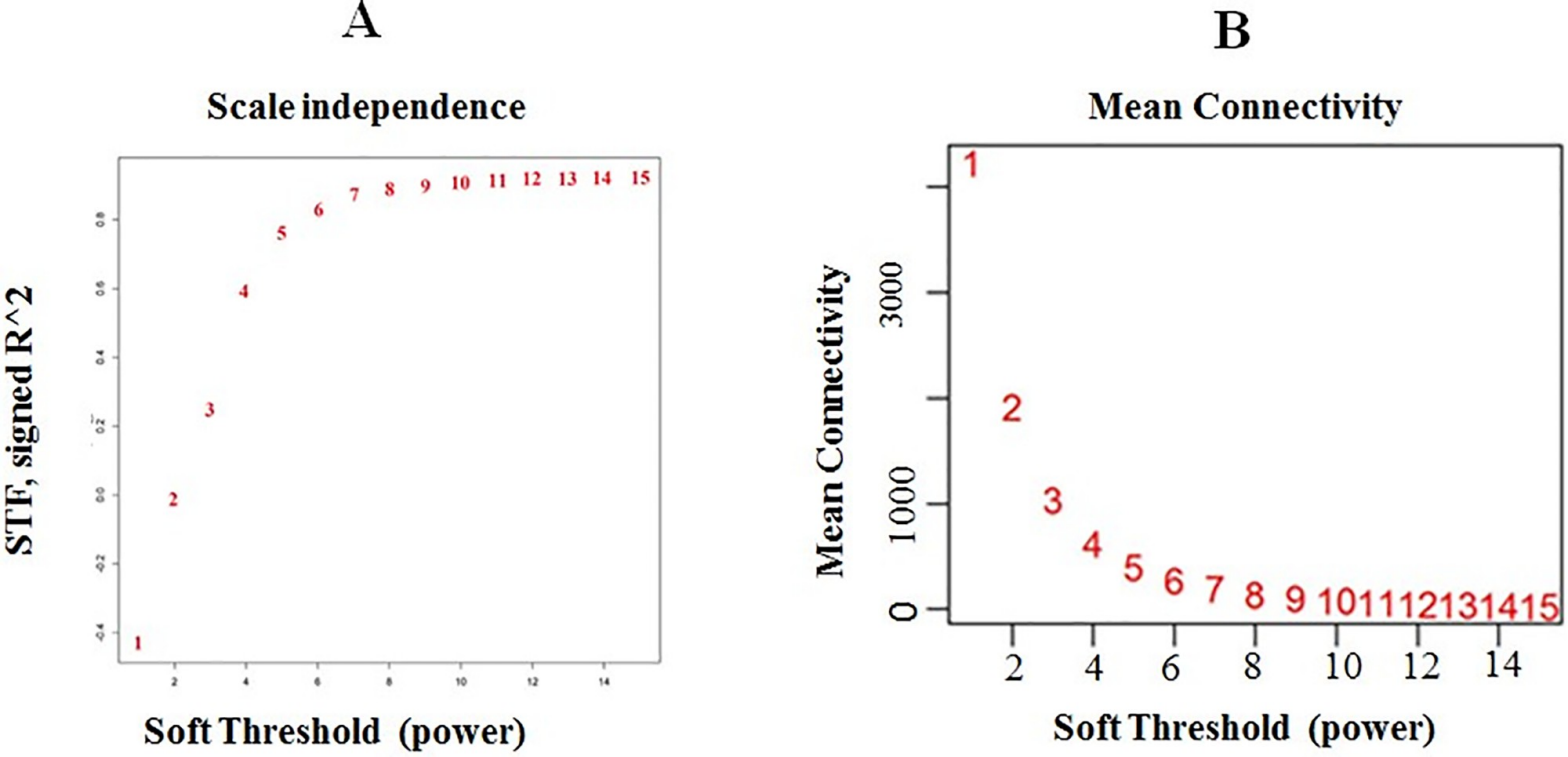
The relationship of Soft Threshold (power) with Scale Free Topology (A) and Mean Connectivity (B). The number showing the power threshold for each soft power corresponding to the SFT and connectivity mean.

**Fig 3:**
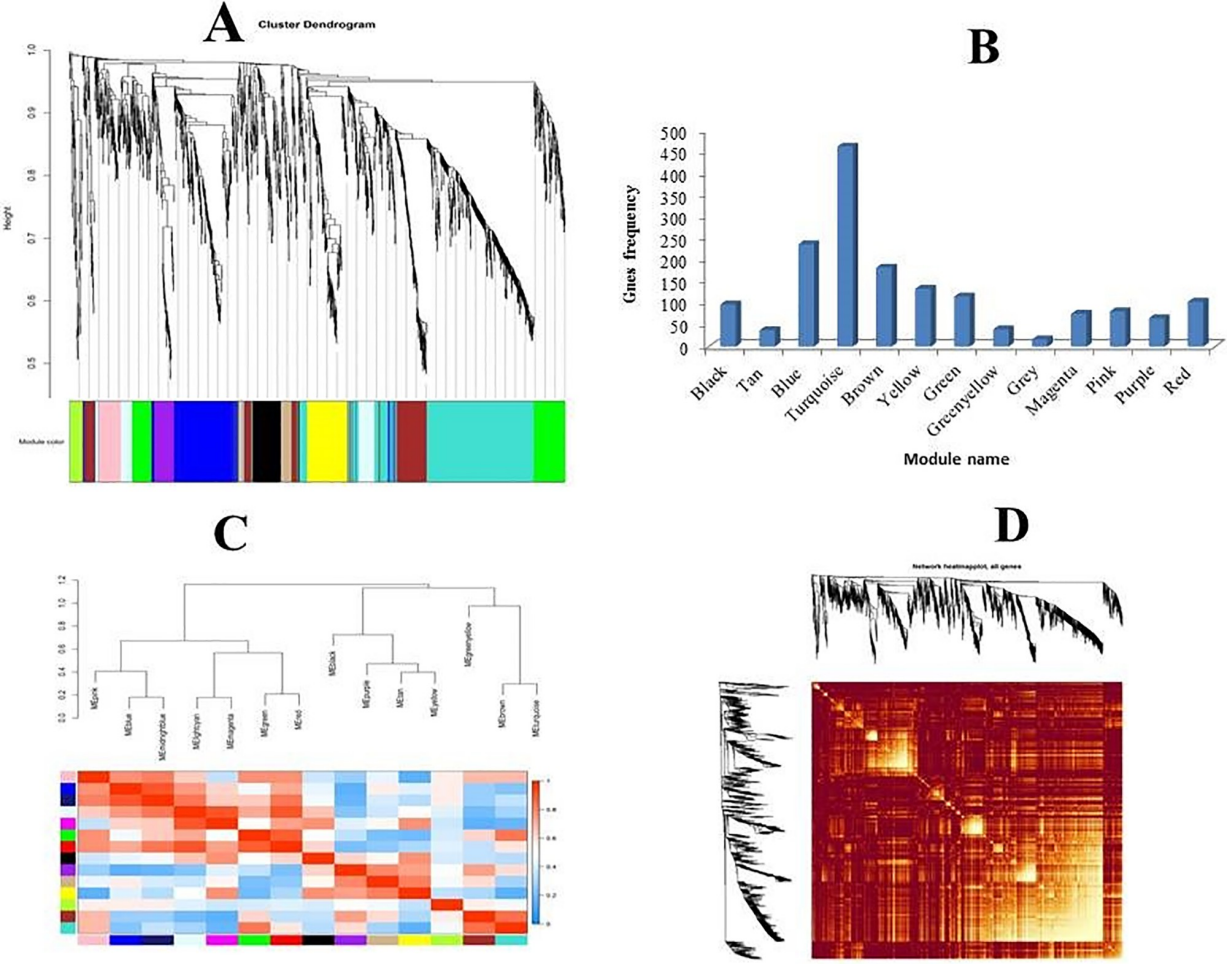
Weighted gene co-expression network analysis of stress responses genes in *Chromochloris zofingiensis*. (A) Determined functional modules based on hierarchical cluster tree of the Meta genes. (B) The sizes of determined modules based on the number of involved genes. (C) The module Eigen gene adjacency estimated by hierarchical clustering and shown by heat map. (B) Topological Overlap Matrix (TOM) value among the proteins of the network delimited in modules by the dynamic method.

The first step for reliable network construction and module detection is the removal of the outlier samples from initial datasets. The results obtained from the preliminary analysis of samples based on the distance-based adjacency metrics showed that two samples of stress treated groups had a standard connectivity score lower than −2.5, which were removed and the remaining samples (55 samples) included for weighted co-expression network construction (Fig 2A). When Soft-Threshold (power) was seven, the Scale Free Topology fitting index (R2) was higher than 0.8 and Mean Connectivity became stable (Fig 2). Therefore, the weighted gene-co expression network was constructed using beta index = 7. In total 12 co-expressed modules were identified with an average size of 128 genes (Fig 3A). Of which, tan and turquoise modules with a size of 463 and 38 genes were the largest and smallest modules, respectively (Fig 3B). The irrelevant genes were allocated to the gray module with 17 genes. Details of each module including the size and genes are provided in S2 Table. Moreover, the heat map shows the topological overlap matrix (TOM) value among the nodes of the network delimited in modules by the dynamic method (Fig 3C). Yellow and progressively red colors indicate the low and higher TOM values, respectively. Expression profiles of each module were summarized as the eigenvector correlated to the first main component of the expression matrix using the module eigengene (Fig 3D).

### Module preservation analysis

**Fig 4:**
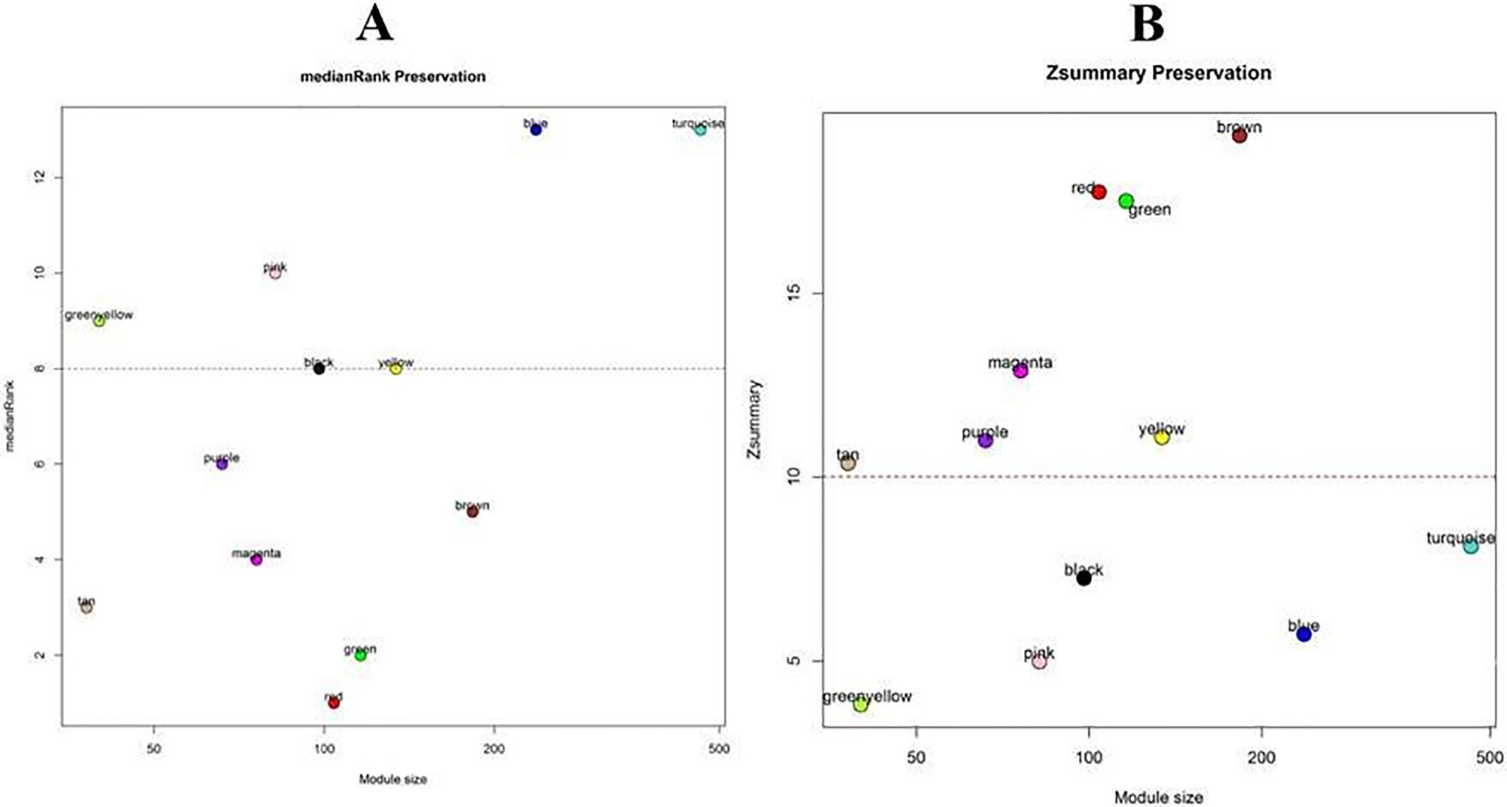
Preservation analysis of co-expressed modules of control networks were tested in the stress condition based on the median rank (A) and Zsummary (B) algorithms. Each dots indicated the corresponding modules which was defined based on WGCNA method.

Identified the non-preserved co-expressed modules as informative modules in stress condition in comparison with the control condition, two medianRankand Zsummary algorithms were harnessed (Fig 4). As can be seen from Fig 4, six modules including the blue, black, yellow, pink, greenyellow, and turquoise were the non-preserved modules. The connectivity and density patterns of the aforementioned non-preserved modules varied in the control and stress conditions. Therefore, we selected these modules for further functional impact analysis.

### Functional enrichment analysis

**Fig 5:**
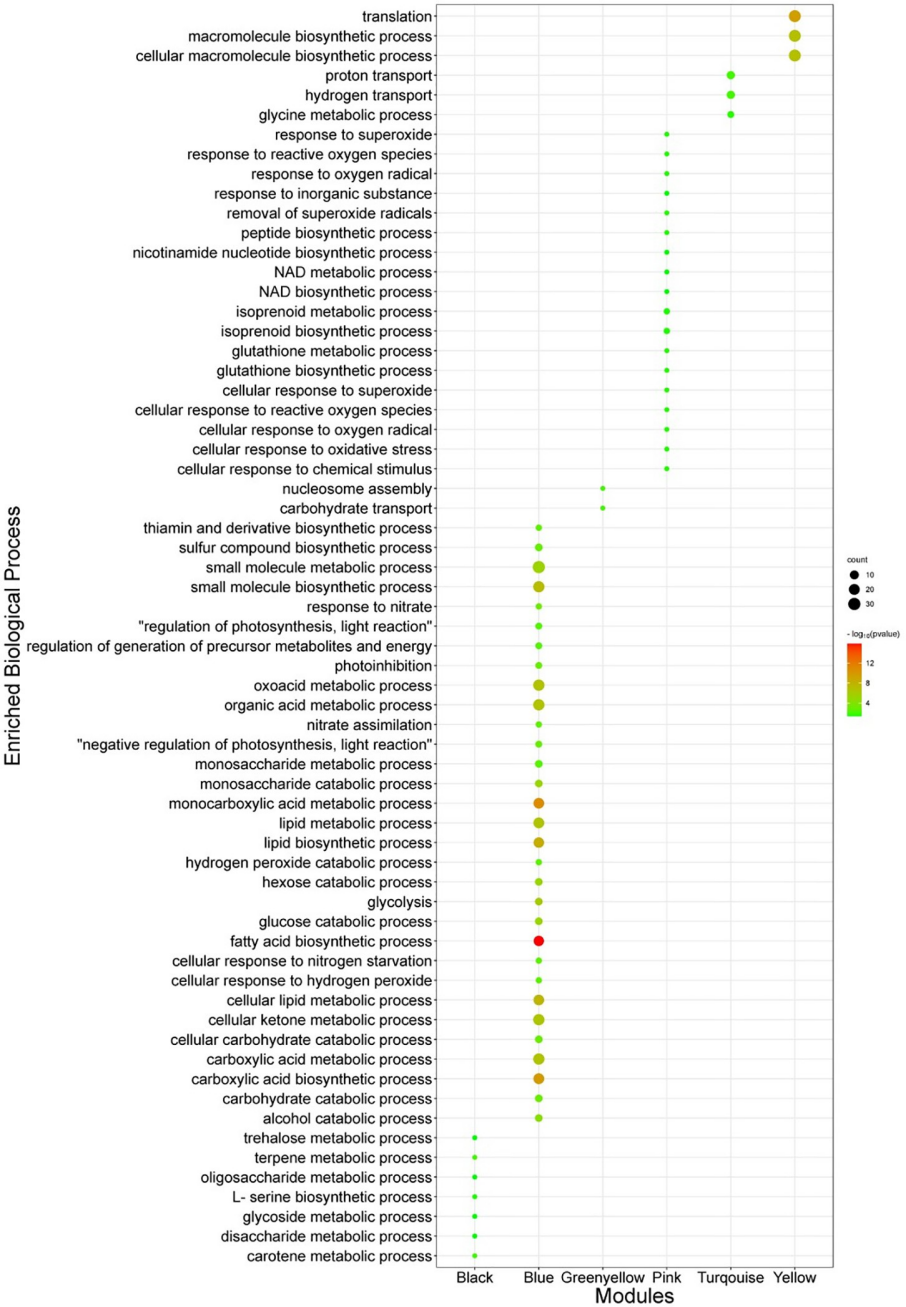
Functional impact of non-preserved modules-based gene ontology (biological processes category).

Functional enrichment analysis of non-preserved modules was performed to shed light on the biological performance of underlying genes in the network context. Functional enrichment based on gene ontology (GO) showed that each of the non-preserved modules was enriched in distinct biological processes and molecular functions. For example, GO terms such as “Trehalose metabolic process” and “Carotene metabolic process” were significantly enriched in the black module (Fig 5). Regarding the turquoise module, “Proton transport” and “Glycine metabolic process” were the most significant enriched GO term in the biological process category (Fig 5). It is whilst; “Nucleosome assembly” and “Carbohydrate transporter” were the most significantly enriched GO term in the biological process category in the greenyellow module (Fig 5). Biological process of “reactive oxygen” and “scavenging related process” were significantly enriched in the pink module. It is whilst the “translation” and “Fatty acid biosynthetic” were the most significantly enriched biological process in the yellow and blue modules, respectively.

The most significantly enriched KEEG pathways of the non-preserved were presented in Table 1. The most significant KEGG pathway in the blue module was “Fatty acid biosynthesis”, “Glycolysis / Gluconeogenesis”, “Pyruvate metabolism”, and “Biosynthesis of plant hormones” (Table 1). It is whilst, the turquoise models enriched in KEGG pathways such as “Cyanoamino acid metabolism”, “Oxidative phosphorylation”, “Vitamin B6 metabolism”, and “Arachidonic acid metabolism”. More details about the enriched KEGG pathways of other non-preserved modules are provided in Table 1.

**Table 1:** The significantly enriched KEGG pathways in non-preserved module under stress condition.

Modules	KEGG pathway	Adj.pvalue
Blue	Fatty acid biosynthesis	8.80E-15
Blue	Glycolysis / Gluconeogenesis	5.44E-09
Blue	Biosynthesis of phenylpropanoids	1.18E-07
Blue	Pyruvate metabolism	2.29E-07
Blue	Tetracycline biosynthesis	2.93E-07
Blue	Biosynthesis of alkaloids	1.74E-06
Blue	Biosynthesis of unsaturated fatty acids	3.78E-06
Blue	Propanoate metabolism	8.66E-06
Blue	Biosynthesis of terpenoids and steroids	1.37E-05
Blue	Biosynthesis of plant hormones	3.40E-05
Blue	Citrate cycle (TCA cycle)	0.000229
Blue	1- and 2-Methylnaphthalene degradation	0.00068
Blue	Phenylpropanoid biosynthesis	0.003606
Blue	3-Chloroacrylic acid degradation	0.003643
Blue	Butanoate metabolism	0.003848
Blue	Tryptophan metabolism	0.006944
Blue	Glyoxylate and dicarboxylate metabolism	0.015043
Blue	Nitrogen metabolism	0.017642
Blue	Metabolism of xenobiotics by cytochrome P450	0.028598
Blue	Drug metabolism—cytochrome P450	0.030399
Blue	Ascorbate and aldarate metabolism	0.043288
Blue	Benzoate degradation via hydroxylation	0.047074
Blue	Fatty acid elongation in mitochondria	0.047074
Black	Methane metabolism	0.00293
Black	Flavonoid biosynthesis	0.024893
Black	Starch and sucrose metabolism	0.037901
Black	Biotin metabolism	0.042317
Turquoise	Amino acid metabolism	0.001281
Turquoise	Oxidative phosphorylation	0.006018
Turquoise	Flavonoid biosynthesis	0.01031
Turquoise	Arachidonic acid metabolism	0.013439
Turquoise	Vitamin B6 metabolism	0.021117
Pink	Biosynthesis of ansamycins	0.004715
Pink	Nicotinate and nicotinamide metabolism	0.005951
Pink	Glycosphingolipid biosynthesis	0.009409
Pink	Circadian rhythm	0.027973
Pink	Terpenoid backbone biosynthesis	0.021117
Pink	Carotenoid biosynthesis	0.023125
Yellow	Ribosome	6.98E-33
Greenyellow	Phenylpropanoid biosynthesis	0.005379
Greenyellow	Amino acid metabolism	0.008954
Greenyellow	Adipocytokine signaling pathway	0.018733
Greenyellow	Pathogenic Escherichia coli infection	0.024918

### TFs and hub genes in non-preserved modules

**Fig 6:**
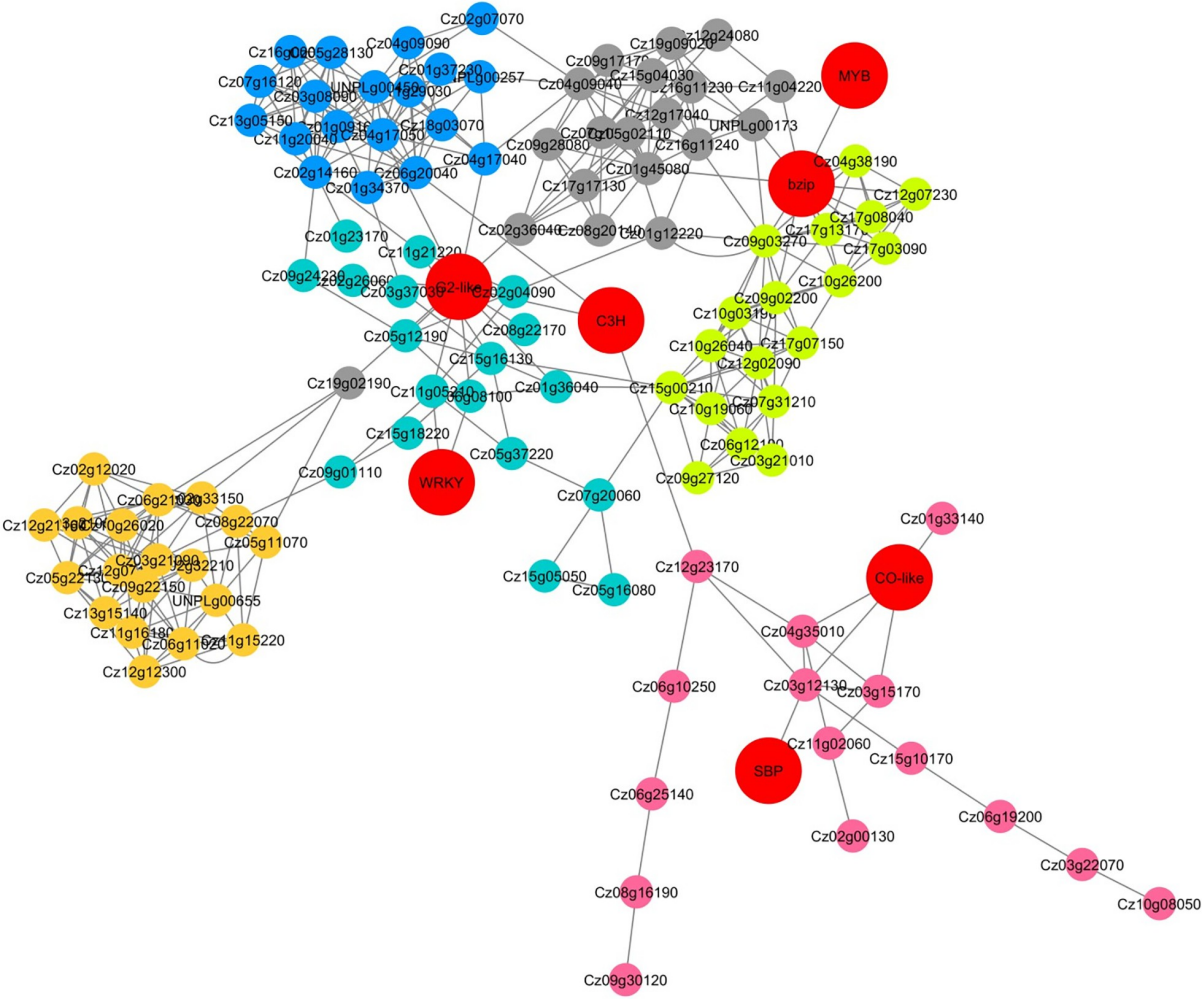
Regulatory networks between Hubs and TFs of non-preserved co-expressed modules under stress condition. Each dots indicated the genes involved in coexpressed modules. Here in we defined the dot as a node in the constructed network. Node colors represent correspondence module color of hub genes. TFs were shown with bigger red nodes.

Annotation of each non-preserved module based on the TFs sequences identifies some TFs in non-preserved modules which are presented in Table 2. It is apparent that the black and blue modules harbored the one bZIP transcription factor. It is whilst the greenyellow contains the MYB TFs. As shown in Table 2, higher numbers of TFs were identified in the turquoise module which belong to B3, SBP, G2-like, bZIP, MYB, AP2, C3H, and WRKY families (Table 2). Using the intramodular connectivity criterion, the hub genes which had critical importance and representative of the module’s overall function were defined in each non-significant module (Fig 6). The top 20 hub genes in each module were presented in the S3 Table. To validate the hub genes discriminative power between stress and un-stressed condition, the LOOCV method was applied to the expression value of hub genes as described in (Panahi and Hejazi, 2021). Results showed that the identified hub genes have distinguished two conditions with 90.18% accuracy, highlighting the discriminative efficiency of identified hub genes and validating the defined hubs.

**Table 2:** Identified transcription factors (TFs) and corresponding non-preserved modules.

Module	Identified TFs	TFs annotation
Black	Cz09g18200	bZIP family
Blue	Cz06g11180	bZIP family
Greenyellow	Cz15g15180	MYB family
Pink	Cz04g06060	CO-like family
Pink	Cz10g15210	C3H family
Turquoise	Cz02g09060	B3 family
Turquoise	Cz02g40170	SBP family
Turquoise	Cz07g32220	SBP family
Turquoise	Cz11g17160	G2-like family
Turquoise	Cz13g00180	bZIP family
Turquoise	Cz14g00040	MYB family
Turquoise	Cz14g11030	AP2 family
Turquoise	Cz17g07180	bZIP family
Turquoise	Cz17g10050	C3H family
Turquoise	Cz19g10020	G2-like family
Turquoise	UNPLg00488	WRKY family
Yellow	Cz06g16250	C3H family
Yellow	Cz10g12010	G2-like family

### TFs- Hub genes regulatory network

As mentioned in the methods and material section, the ensemble of tree algorithms with the number of trees = 1000 was harnessed for regulatory network inference between TFs and top hub genes in non-preserved modules (Fig 6). It was shown that the transcription factor CO-like, C3H, and SQUAMOSA promoter binding protein (SBP) specifically regulates the hub genes of the pink module. Cz03g12130 and Cz01g37230 genes encode the elongation factor EF-3, and pyruvate dehydrogenase E1 beta subunit, respectively, directly regulated by the SBP TF. It is whilst that the MYB TFs directly regulate the Cz01g45080 gene which encodes the HAC1 transcription factor involved in signaling from the endoplasmic reticulum to the nucleus under stress conditions. WRKY and other TFs play an important role in the constructed regulatory networks, directly interacting with the UNPLg00655 gene which encodes RPL18a protein (60S ribosomal protein L18a). Moreover, G2-like TFs were directly interacting with the Cz19g09020 gene which encodes the lipid kinase YegS-like protein (Fig 6). C3H transcription factor directly interacts with Cz12g23170 gene encoding ribosome biogenesis protein BOP1 and affects the pink functional module (Fig 6).

## Discussion

The results showed that the topological features of the co-expression network of stress-responsive genes varied under stress conditions in comparison with control conditions and these genes created some co-expressed modules with distinct functional enrichments (Figs 4 and 5). Preservation analyses with permutation tests were further performed to compare the connectivity pattern of genes involved in these co-expressed modules between control and stress conditions, leading to identify non-preserved co-expressed modules with significant underlying pathways and hubs which may be the backbone of stress underpinning mechanisms of *C*. *zofingiensis*. As mentioned in the results section, five modules including black, blue, yellow, greenyellow, and pink were defined as none-preserved modules and were considered for further discussion.

The black module was significantly enriched in methane metabolism, starch and sucrose metabolism, and biotin metabolism. The contribution of methane metabolism in stress-responsive mechanisms are reported by different studies [40, 41]. Two UNPLg00177 and Cz02g17100 genes encoding the S-(hydroxymethyl) glutathione dehydrogenase and catalase showed the connectivity pattern reforming during stress conditions. Catalase also contributed to the MAPK signaling pathway, FoxO signaling pathway, tryptophan metabolism, and the topological variation of catalase interaction highlighted the role of this protein in the synchronization of stress responsive pathways at systems levels. Starch and sucrose metabolism are other pathways which are significantly enriched in the black module (Fig 5 and Table 1). Along with the results of this study, it has been shown that the starch metabolisms were up-regulated under sulfur starvation, nitrogen starvation, and salt stress condition [42]. In the stress condition, protein synthesis was inhibited, which resulted in the reallocation of carbon and up-regulation of the starch synthesis pathway by increasing the expressions of glycogen branching and starch synthase enzymes. Among different genes involved in the starch and sucrose metabolism with similar expression patterns, the connectivity pattern of Cz09g30050 and Cz05g05080 genes which encode the alpha-trehalase and fructokinase, respectively, were changed at stress conditions. Alpha-trehalase is catalysis the hydrolysis of trehalose, as a potential signal metabolite in energy metabolism. It has been demonstrated that trehalose regulates starch and energy metabolism to enhance tolerance under stress conditions [43]. Dissection of regulatory networks of identified TFs also showed that the bZIP (basic leucine zipper) family is among the most important transcriptional regulators of the enriched pathways in the black module of *C*. *zofingiensis* under stress conditions (Fig 6). In line with our findings, the contribution of bZIP transcription factor in *chlorella* microalgae responses to the nutrient deficient condition is reported by [44–46]. Moreover, bZIP transcription factors contribution to the induction of the carbon-concentrating mechanism at oxidative stress conditions has been proven [47]. More interestingly, bZIP Transcription Factor HAC-1 were among the hubs/essential genes with higher modular intra modular connectivity in black module which highlighted the contribution of this TF family in transcriptional regulation of methane metabolism, starch and sucrose metabolism, and biotin metabolism during stress condition (Fig 6, S3 Table).

Significant functional terms such as “Glycolysis / Gluconeogenesis”, “Pyruvate metabolism”, “Glyoxylate metabolism”, and “Citrate cycle (TCA cycle)” demonstrated that the blue module was closely related to central carbon metabolism (Fig 5, Table 1). It has been shown that the stress condition leads to redistribution of carbon sinks through the central carbon metabolism regulation [48]. In line with our results, a prior study demonstrated that the excess light and nitrogen starvation shift the carbon metabolisms to increase the pyruvate availability [48]. Our results also confirmed that the carbon metabolisms underlying genes not only respond at the expression level, but also their connectivity pattern at stress condition was changed. This topological change of carbon metabolisms involving genes may be an important key point for efficient regulation of carbon levels. Especially, it has been proven that the increased carbon availability has an expense for cell growth and viability under stress conditions [49, 50]. More dissection of top hub genes in the blue module, it was shown that the Cz04g17050 (PYK5), Cz16g00040 (PYK1), Cz01g37230 (PDH2), and Cz03g08090 (PDC2) genes which encode pyruvate kinase, pyruvate kinase, putative pyruvate dehydrogenase E1 beta subunit, and plastid pyruvate dehydrogenase E1 alpha subunit, respectively, were among the hubs with the highest number of intramodular connectivity, highlighting the importance of these genes in central carbon metabolism network remodeling during stress condition. It has been shown that the pyruvate kinase up-regulation increases the glycolysis pathways. This up-regulation aids the conversion of photosynthetic carbon to pyruvate as an imperative precursor for bio pigments such as astaxanthin and lipid synthesis [13, 50, 51]. Moreover, the analysis showed that the expression and connectivity of central carbon metabolism and fatty acid biosynthesis metabolisms underlying genes were coordinately changed during stress conditions. What is interesting is that the UNPLg00257 (KAS2) which encodes the β-Ketoacyl-acyl-carrier-protein synthase III and is involved in the dissociated (or type II) fatty-acid biosynthesis system were among the hub genes of this module and coordinately expressed with central carbon metabolism related hub genes. The regulatory network also deciphered that the bZIP and CO-like TFs are among the most important transcriptional regulators of central carbon metabolism and fatty acid biosynthesis pathways during stress conditions. Nevertheless, bZIP were among the co-expressed genes of blue, however, constructed regulatory networks based on ensemble tree methods showed that the CO-like TFs directly regulate this module (Fig 6).

Functional analysis of the yellow module showed that this module was enriched in the “Ribosome” and “Translation” KEGG pathway and biological process, respectively (Table 1, Fig 5). Additionally, some important hub-high traffic genes involved in the translation during stress conditions were Cz06g21030, Cz03g21090, Cz13g15140, Cz05g22130, Cz12g21160, Cz12g12300, and Cz12g07160 which encode the RPL15, RPL14, RPS24, RPL13a, RPS14, RPL35, and RPS9 proteins, respectively (S3 Table). Translation machinery (ribosome subunits) modulation at expression level in different microalgae has been reported by prior studies under stress conditions. It has been demonstrated that the ROS producing stresses trigger redox-based posttranslational mechanisms after the modulation of translation machinery [52]. Moreover, these translation-related proteins were co-expressed with C3H and G2-like TFs which were further confirmed by the constructed TF-Hub genes network (Table 2 and Fig 6).

KEGG pathways and biological processes analysis of the turquoise module revealed that the co-expressed genes of this module were highly enriched in pathways such as “Oxidative phosphorylation”, “Amino acid metabolism”, “Arachidonic acid metabolism”, and “Vitamin B6 metabolism” as well as biological process such as “proton transport”, “hydrogen transport”, and “glycine metabolic process” (Table 1 and Fig 5). It is known that photosynthesis and oxidative phosphorylation are essential for the production of ATP [53]. Prior study on *Chlamydomonas* showed that nitrogen deprivation up-regulated the oxidative phosphorylation related genes, such as NADH dehydrogenase, ATP synthases, and cytochromec oxidase [54]. Our findings also revealed that the genes encoding the H^+^-transporting ATPase, V-type H^+^-transporting ATPase, cytochrome c oxidase, F-type H+-transporting ATPase, inorganic pyrophosphatase, H^+^-, coordinately regulated under stress condition. Such results have been also reported previously [55]. Moreover, our results showed that the connectivity pattern of the mentioned genes was coordinately varied under stress conditions. It was found that the glycine hydroxyl methyl transferase (SHMT) gene had an important function in the regulation of amino acid metabolism of *C*. *zofingiensis* under stress conditions. The importance of SHMT connectivity reforming during salt stress conditions was also previously suggested in halophyte microalgae *Dunaliella salina* [2, 20]. Vitamin B6 metabolism was among the most significantly enriched pathways in the turquoise module. Vitamin B6 (pyridoxine, PN) and its vitamer derivatives pyridoxal by their ability to function as ROS neutralizing had a pivotal role behind the stress tolerance [56, 57]. Among different genes involved in vitamin B6 metabolism, phosphoserine aminotransferase and pyridoxal 5’-phosphate synthase showed the coordinated expression and connectivity pattern with oxidative phosphorylation related genes, indicating the importance of the mentioned genes function under stress conditions. As shown in the Table 2, annotation and screening of genes involved in the turquoise module suggested that the B3, SBP, G2-like, bZIP, MYB, AP2, C3H, G2-like, and WRKY TFs contribute in transcriptional regulation of the Oxidative phosphorylation”, “Amino acid metabolism”, “Arachidonic acid metabolism”, and “Vitamin B6 metabolism” under stress condition. The contribution of MYB TFs in vitamin B6 metabolism regulation during the transition of algae from autotrophic to heterotrophic growth condition was shown in a previous study [35]. Constructed regulatory network of top hub genes in turquoise module and the mentioned TFs further highlighted the regulatory impact of the mentioned TFs in aforementioned metabolisms (Fig 6).

In the pink module, co-expressed genes were enriched in the “Biosynthesis of ansamycins”, “Nicotinate and nicotinamide metabolism”, “Glycosphingolipid biosynthesis”, “Terpenoid backbone biosynthesis”, and “Carotenoid biosynthesis” pathways (Table 1). Gene encoding the transketolase was the key gene in the biosynthesis of ansamycins, which connectivity pattern was changed during the stress condition. Moreover, our analysis confirmed the functional importance of the gene Cz04g35010 encoding the 9-cis-epoxycarotenoid dioxygenase protein which is related to the carotenogenic pathway under the overall stress conditions in both expression and systems levels. Network analysis also highlighted the regulatory impacts of CO-like, C3H, and SBP TFs on the biosynthesis of ansamycins and carotenogenic pathways during stress conditions.

More additionally, our study dissected the regulators of high-value compounds during stress condition. Results showed that the TFs such as NF-Y, WRKY, and MYB have been implicated in the regulation of lipid metabolism in *C*. *zofingiensis* under multiple stress condition. In line with our findings, molecular mechanisms and functions of CzMYB1 in response to TAG-inducing conditions and correlation of these genes with the de novo fatty acid synthesis, fatty acid activation and desaturation, membrane lipid turnover, and TAG assembly involved genes in *C*. *zofingiensis* has been previously demonstrated [58], highlighting the expression and connectivity rewiring of these TFs under TAG-inducing conditions. Moreover, we found that the TFs such as NAC and APETALA2/Ethylene-Responsive Factor (AP2/ERF) significantly correlated with astaxanthin biosynthesis related genes. The correlation between TFs and astaxanthin-related genes serves as strong evidence for the regulatory functions of these TFs in astaxanthin accumulation. correlation between DpAP2 and carotenoid accumulation was previously reported in Marine Microalga *Dunaliella parva* [59]. In conclusion, the integrative approach which was used in this study revealed the important metabolisms and pathways in which expression and connectivity patterns of involved genes were changed under stress conditions. Furthermore, we delineated the most important hub genes of enriched pathways of non-preserved modules to construct the regulatory networks with identified TFs using a machine learning model. Our analysis highlighted the functional importance of central carbon metabolisms, fatty acid metabolisms, vitamin B6 metabolisms, etc. in responding to different stress conditions. Moreover, this study provides valuable information regarding the regulatory impacts of different TFs in different non-preserved related hubs. The current findings add substantially to our understanding of the stress responsive underlying mechanism of *C*. *zofingiensis* which can serve as a base for future studies and different metabolite production optimization programs.

## Supporting information

S1 Table The list of genes which were identified after direct merging. (XLSX)

S2 Table Details of modules and their involved genes. (XLSX)

S3 Table Top 20 hub genes identified in non-preserved module based on module intraconnectivity. (XLSX)
